# Supplementary material for: Identification of Freezing-Responsive microRNAs and Their Targets in Chinese Jujube by Small RNA and Degradome Sequencing
Source: Int J Mol Sci. 2026 Sep 4;27(17):7912. doi: 10.3390/ijms27177912 (PMC13566890; doi:10.3390/ijms27177912)
Supplement: Supplementary file 1 [file ijms-27-07912-s001.zip › Supplementary Material S2/novel_miR_24.pdf]

Provisional ID	:	novel_miR_24
Score total	:	0.4
Score for star read(s)	:	-1.3
Score for read counts	:	0
Score for mfe	:	0.1
Score for randfold	:	1.6
Score for cons. seed	:	
Total read count	:	94
Mature read count	:	94
Loop read count	:	0
Star read count	:	0

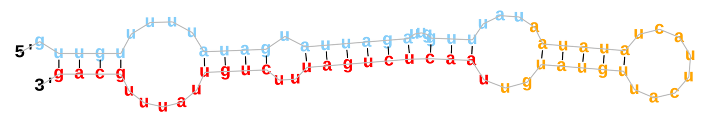

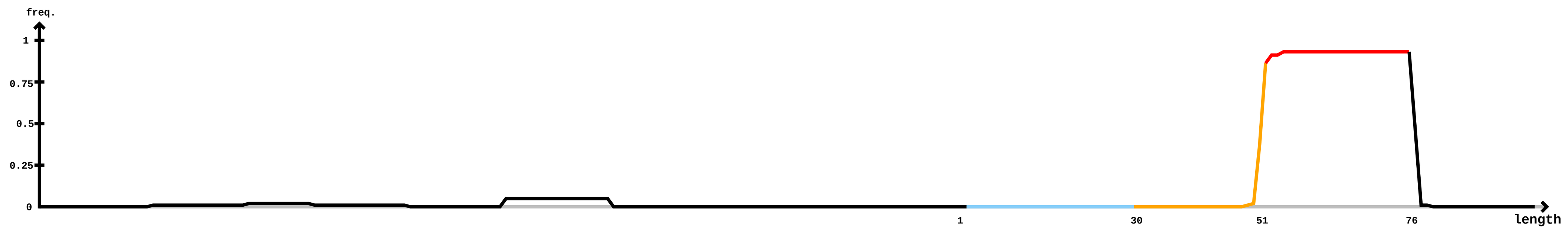

Star Mature

```
5'- cauuuguuugucacauucauugagaaguacugauucugacggaggacauguguaugugcauuauauagaguuauaaugaacuuguuuacuuugucauuaaauguugcuuguguuaaauaauuuuucuuuucaguugguaguucucaucuagcuguuguuuuuauaguauuagauugguuuauaauauaucauucauuguauguuaacucugauuucuguuauuugcagcagaugaaaaucuuccugccaa -3'   exp
    ...(((((((.((((..(((((((((......)))((((((((((((.((.......(((((((.....))))))).)).)))).))))))))))))).....)))).)))))))............((((((((.((((((.(((((.......((((.((((((...(((....(((((........)))))...)))))))))..)))).....))))))))))).)).....)))))))   reads   mm   sample
```

Aligned read	reads	mm	sample
................cauugagaaguacugauucugacggag....	1	0	LCK-3
................................uucugacggaggacauguguaugugca....	1	0	JT-3
....ugaUcuuguuuacuuugu....	3	1	JT-2
....ugaUcuuguuuacuuugu....	2	1	JT-3
....uguuaacucugauuucuguuauuugcag....	1	0	LT-3
....guuaacucugauuucuguuauuugcag....	1	0	LT-3
....uuaacucugauuucuguuauuugcag....	6	0	LT-1
....uuaacucugauuucuguuauuCgcag....	1	1	JCK-2
....uuaacucugauuucuguuauuugcag....	7	0	JCK-3
....uuaacucugauuucuguuauuugcag....	5	0	JCK-1
....uuaacucugauuucuguuauuugcag....	13	0	JCK-2
....uuaacucugauuucuguuauuugcag....	1	0	LT-3
....uuaacucugauuucuguuauuugcag....	2	0	LT-2
....uuaacucugauuucuguuauuugcagcag....	1	0	JCK-3
....uaacucugauuucuguuauuugcNg....	1	1	LT-2
....uaacucugauuucuguuauuugcag....	2	0	LT-2
....uaacucugauuucuguuauuugcag....	4	0	LT-3
....uaacucugauuucuguuauuugcag....	7	0	LCK-3
....uaacucugauuucuguuauuugcag....	5	0	JCK-1
....uaacucugauuucuguuauuugcag....	5	0	JCK-3
....uaacucugauuucuguuauuugcag....	16	0	JCK-2
....uaacucugauuucuguuauuugcag....	10	0	LT-1
....aacucugauuucuguuauuugcag....	2	0	LT-1
....aacucugauuucuguuauuugcag....	1	0	JCK-2
....aCcucugauuucuguuauuugcag....	1	1	JCK-3
....aacucugauuucuguuauuugcag....	1	0	JCK-1
....cucugauuucuguuauuugcag....	2	0	LCK-3
